# Danhong Promotes Angiogenesis in Diabetic Mice after Critical Limb Ischemia by Activation of CSE-H_**2**_S-VEGF Axis

**DOI:** 10.1155/2015/276263

**Published:** 2015-09-30

**Authors:** Feng Wu, Zhiqing He, Ru Ding, Zhigang Huang, Qixia Jiang, Haiming Cui, Yi Lin, Shuaibo Huang, Xianliang Dai, Jiayou Zhang, Zonggui Wu, Chun Liang

**Affiliations:** ^1^Department of Cardiology, Shanghai Changzheng Hospital, Second Military Medical University, No. 415, Fengyang Road, Huangpu District, Shanghai 200003, China; ^2^Department of Cardiology, Fuzhou General Hospital of Nanjing Military Command, No. 156, Xi Erhuan North Road, Fuzhou, Fujian 350025, China

## Abstract

The aim of this paper is to investigate effect and mechanism of Danhong injection (DH) on angiogenesis in the diabetic hind limb ischemia mouse model. Thirty diabetic hind limb ischemic model mice and ten normal mice, established by intraperitoneal (i.p.) injection of streptozotocin (STZ) or PBS and ligation/excision of femoral artery, and then twenty diabetic hind limb ischemic model mice of all were evenly randomized to saline (control, *n* = 10) and DH i.p. injection (2 mL/kg weight for 7 days, *n* = 10) groups. Limb perfusion recovery and femoral blood hydrogen sulfide (H_2_S) and vessel regeneration and lower limb vascular endothelial growth factor (VEGF)/cystathionine *γ*-lyase (CSE) expression were evaluated during intervention and after euthanasia, respectively. DH i.p. increased ischemic limb perfusion and promoted collateral circulation generation without decreasing blood glucose level. Increased local CSE-H_2_S-VEGF expression contributed to beneficial effects of DH injection. In conclusion, activation of local CSE-H_2_S-VEGF axis might participate in proangiogenesis effects of DH injection in diabetic hind limb ischemia model mice, suggesting a potential therapy for diabetic patients with critical limb ischemia.

## 1. Introduction

Current standard of care for critical limb ischemia (CLI), annually affecting estimated 500 to 1000 per million people worldwide [1] and particularly diabetics, includes lifestyle modification, pharmacotherapy to reduce blood cholesterol, glucose, and hypertension, and revascularization by angioplasty or bypass surgery. However, revascularization is associated with high long-term restenosis rate, does not address underlying pathology, and is not an option for all patients [2], who often undergo major limb amputation with 30% second amputation and 25% death rates within one year [3] prompting continued search for clinical and cost-effective treatments. Among traditional Chinese medicine (TCM) drugs potentially useful for therapeutic angiogenesis, Danhong injection (DH), a Chinese Materia Medica standardized product extracted from* Radix Salviae miltiorrhizae* and* Flos Carthamus tinctorius*, [4], has been shown in animal and clinical studies to improve angina and decrease acute and chronic cardiovascular event occurrence by modulating angiogenesis, inflammation, immunity, and oxidative stress [5–10]. This study therefore examined proangiogenesis effects and underlying mechanisms of Danhong injection in the diabetic hind limb ischemia mouse model to gain insight for potential clinical use in diabetics with CLI.

## 2. Materials and Methods

### 2.1. Preparation of DH and Quality Control

According to the production protocol of DH injection provided by Shanxi Buchang Pharmaceutical Co. (Shanxi, China), powdered* Radix et Rhizoma Salviae miltiorrhizae* (750 g) is twice immersed in 7.5 L of 30% ethanol in order to maximally dissolve active ingredient, and then both of the immersions are extracted for 1 h at 50°C, after filtration, and mixed with* Flos Carthamus* (250 g). The mixture is then twice immersed in 2.5 L of water for 1 h at 35°C and vacuum evaporated to relative density of 1.10–1.20 (65°C). The solution is filtered and stored at 4°C for 24 h. Water is added to the solution of 1.0 L; sodium chloride and sodium hydroxide are added to achieve an isotonic solution with pH of 6-7 for injection, again filtered, and then sterilized and encapsulated into ampoules (10 mL per ampoule). DH was approved over 5 years ago by the Chinese Food and Drug Administration (CFDA) as Chinese herbal patented product for coronary heart disease patients and listed in the Chinese Pharmacopoeia (Heze Buchang Pharmaceutical Co., Ltd., drug approval number Z20026866).

DH contains two herbal medicinal components,* Salvia miltiorrhiza BUNGE* and* Carthamus tinctorius L*, authenticated and standardized based on marker compounds according to Chinese Pharmacopoeia 2005. DH dose variability was minimized by strict standardization of batches, species, origin, harvest time, medicinal components, and preparation methods, which was confirmed by high performance liquid chromatography (HPLC) according to established protocol [11].

### 2.2. Chemicals and Reagents

Purified rat anti-mouse CD31 monoclonal IgG2a antibody was purchased from BD Bioscience (San Diego, CA, USA). Rabbit anti-mouse vascular endothelial growth factor (VEGF) polyclonal antibody was from Santa Cruz Biotechnology (Santa Cruz, CA, USA). Anticystathionine *γ*-lyase (CSE), beta-actin antibodies, and horseradish peroxidase-linked secondary antibodies were obtained from Abcam (San Francisco, CA, USA). Mouse VEGF ELISA kits were purchased from eBioscience (San Diego, CA, USA). Sodium sulfide standard was from Alfa Aesar (cat. number 65122, Ward Hill, MA, USA). Streptozotocin (STZ) and other chemicals frequently used in our laboratory were purchased from Sigma-Aldrich Co. (St Louis, MO, USA).

### 2.3. Diabetic Hind Limb Ischemia Model with BALB/c Mice

**Figure 1:**
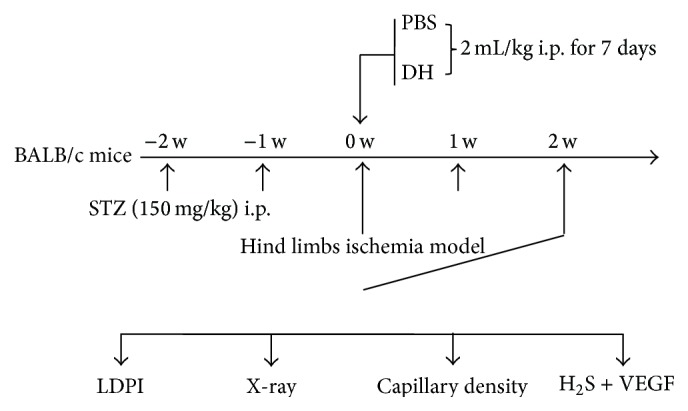
Study protocol. BALB/c mice in hind limb ischemia diabetic model were divided into saline (control, *n* = 10) and DH i.p. injection (2 mL/kg body weight for 7 days, *n* = 10) groups. Diabetic model was established with STZ (150 mg/kg) i.p., and blood glucose levels were monitored for 2 weeks. Diabetes modeling success was identified by twice random blood glucose levels >16 mmol/L. Hind limb ischemia model was established by ligation and excision of femoral artery, saphenous arteries, circumflex of external iliac artery, and muscular branches of femoral artery. Lower limb perfusion was assessed using LDPI. Vein blood was sampled for H_2_S analysis by HPLC-FLD, mice were euthanized, and collateral circulation was assessed by angiography, digital X-ray imaging, and immunohistochemical staining. CSE and VEGF expression (key in proangiogenic axis) were investigated with qPCR assay.

**Figure 2:**
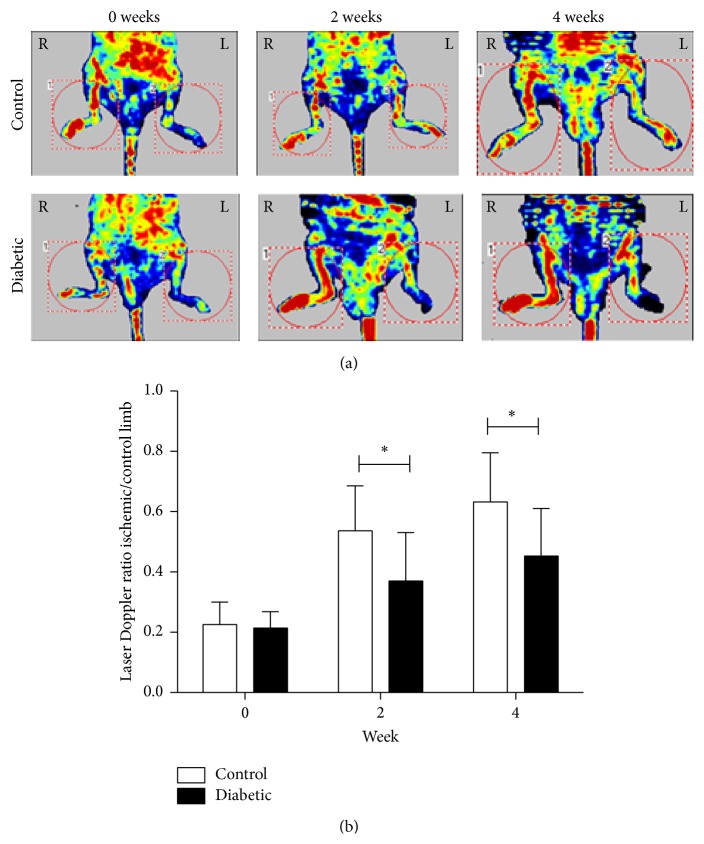
Recovery of blood flow in control and diabetic mice monitored by LPDI. Representative evaluations of ischemic (right) and nonischemic (left) hind limbs, immediately after femoral artery ligation and at weeks 0, 2, and 4. Red and blue indicate normal perfusion and marked blood flow reduction, respectively, in ischemic hind limb. Blood flow recovery (ischemic-to-contralateral hind limb perfusion ratio) is impaired in diabetic versus control mice (nondiabetic mice). Data are expressed as mean ± SD. *∗* indicates *p* < 0.05 versus control.

Study protocol (Figure 1) was approved by Second Military Medical University's Animal Care and Use Committee. After intraperitoneal (i.p.) injection of STZ (150 mg/kg) in BALB/c mice, blood glucose levels were continuously monitored for 2 weeks with diabetes modeling success verified when two random blood glucose levels >16 mmol/L were confirmed. At 2 weeks, hind limb ischemia model was established by ligation and excision of left femoral artery, saphenous arteries, circumflex branch of external iliac artery, and muscular branches of femoral artery [12]. Lower limb perfusion was assessed by laser Doppler perfusion imaging (LDPI) as described below. Immediately after femoral artery ligation, blood flow in the ischemic hind limb was equally reduced in both control and diabetic mice. Consistent with previous studies [13, 14], LDPI showed significantly attenuated perfusion recovery in diabetic compared to control mice on postoperative weeks 4 and 6 (Figures 2(a) and 2(b)), confirming successful STZ-based diabetes and hind limb ischemia modeling of nude mice.

### 2.4. In Vivo Assessment of Limb Function

Semiquantitative assessment of ischemic limb function was performed serially using the following scoring system: 6, full and fast walking; 5, normal but slow walking; 4, walking with only mild deficit; 3, supporting weight, probability of taking 1 or 2 steps; 2, frequent and vigorous movement, no weight bearing; 1, barely perceptible movement, no weight bearing; and 0, no movement [15, 16]. Two independent observers blinded to the study evaluated scores.

### 2.5. Laser Doppler Perfusion Imaging

Mice were anesthetized with intraperitoneal injection of ketamine (60 mg/kg) and xylazine (8 mg/kg). Serial, noninvasive assessment of ischemic limb microvascular perfusion was performed in triplicate and in a blinded manner using a LDPI system (PeriScan PIM 3, Perimed, Sweden) after placing mice on a homoeothermic heating pad maintained at 37°C. Using LDPI image processing software (v5.0), perfusion was quantified in regions, equal in area, encompassing the distal leg (entire foot) of both ischemic and contralateral nonischemic limbs. All perfusion data were expressed as a ratio of operated ischemic to nonoperated control limb perfusion, which could minimize data variation possibly secondary to ambient temperature changes.

### 2.6. HPLC-FLD Analysis of H_2_S in Femoral Artery Blood

Animals were anesthetized with sodium pentobarbital (40 mg/kg body weight intraperitoneal injection), and a 30 G insulin syringe was inserted into the femoral vein to draw blood for H_2_S analysis. H_2_S detection was performed in duplicate for each blood sample following a modified protocol from a previous study based on the fluorescence derivation between monobromobimane (MBB) and hydrogen sulfide (H_2_S) contained in plasma [17]. Briefly, 30 *μ*L plasma was collected after centrifuging and incubated with excess MBB in 100 mM Tris-HCl buffer (pH 9.5, 0.1 mM diethylenetriamine pentaacetic acid) for 30 min in 1% oxygen at room temperature to form a stable derivation, and then the fluorescent product sulfide-dibimane (SDB) was analyzed by RP-HPLC using a Dikma-C18 Leapsil column (2.7 *μ*m × 4.6 mm × 100 mm) with gradient elution by 0.1% (v/v) trifluoroacetic acid in acetonitrile. Using the modified protocol with new HPLC column suitable for both HPLC and UPLC system, detection time could be decreased from 12 to 5 minutes without compromising sensitivity and specificity. Standard curve was established based on different concentrations of sodium sulfide solutions prepared in a strict-control hypoxic chamber by purging with nitrogen gas to 1% O_2_. Retention time of SDB was 3.3 min, and detection limit was 0.5 pM.

### 2.7. Angiographic Assessment of Collateral Circulation

Animals were anesthetized as described above; the hearts were rapidly excised and retrogradely perfused via abdominal aorta with heparin saline (0.1% heparin in 0.9% saline). Post-mortem angiography was performed using Omnipaque (Amersham), hand infused angiographic contrast at 0.5 mL/s for 20 seconds, and a high-definition digital X-ray system (MX-20, Faxitron, USA). Recorded images of the pelvis and both hind limbs were analyzed using Image J software (NIH) to calculate angiographic score of thigh-hip joint to knee area. Specifically, a grid was laid over an image of the arterial filling phase vasculature and number of collateral vessel intersections with the grid counted over a defined, bilaterally equal area. Angiographic score was expressed as ratio of numbers of collateral vessels within operated ischemic-to-contralateral leg.

### 2.8. Histological Assays

After euthanasia, thigh muscles were isolated from limbs and routinely fixed overnight in 4% buffered formalin and embedded in paraffin. Four-micrometer tissue sections were subjected to immune-peroxidase biotin-avidin reaction using the labeled streptavidin biotin method to determine CD31 and VEGF expression. Sections for immunohistochemical analysis were cut and mounted on 3-aminopropyltriethoxysilane-coated (Sigma) slides, allowed to dry overnight at 37°C to ensure optimal adhesion, dewaxed, rehydrated, and treated with 0.3% H_2_O_2_ in methanol for 10 min to block endogenous peroxidase. For antigen retrieval, sections were microwave-treated in 1 mmol/L EDTA at pH 8 for 10 min and then allowed to cool for 20 min. Endogenous biotin was saturated using a biotin blocking kit (Vector Laboratories). Sections were incubated at 37°C for 30 min with the following antibodies: purified rat anti-mouse CD31 (dilution 1 : 30; monoclonal IgG2a, BD Bioscience) and rabbit anti-mouse VEGF (dilution 1 : 100, polyclonal, Santa Cruz Biotechnology). Binding was visualized using biotinylated secondary antibody (1 h incubation) and streptavidin-biotin peroxidase complex developed with diaminobenzidine. Finally, slides were counterstained with hematoxylin. Capillary density and leukocyte infiltration expressed as number of CD31^+^ cells per square millimeter were measured by counting six random high-power (magnification ×200) fields for a minimum of 200 fibers from each ischemic and contralateral limb. The area was measured with Image J software. Two operators analyzed the results independently.

### 2.9. qPCR Assay of CSE-VEGF Axis

RNA was isolated from ischemia hind limbs using the RNeasy kit (Qiagen, Hilden, Germany). Total RNA was analyzed by Nanodrop (Thermo, USA). Reverse transcription was performed with PrimeScript 1st Strand cDNA Synthesis kit (Takara, Japan) and cDNA amplified by iQ SYBR Green Real-Time PCR Supermix (Bio-rad, USA) using primers for VEGF and glyceraldehyde 3-phosphate dehydrogenase (GAPDH) in an CFX Connect real-time PCR detection system from Bio-rad. All primers were obtained from Life Technology (USA). Data were analyzed based on relative expression method with the formula 2^−ΔΔCT^, where ΔΔCT = ΔCT (sample) −  ΔCT (calibrator = average CT values of all samples within each group), with ΔCT being CT of housekeeping gene (GAPDH) subtracted from CT of target gene.

### 2.10. Statistical Analysis

Data are presented as mean ± standard deviation and were compared using paired *t*-test with GraphPad Prism 5.01 (La Jolla, CA, USA) and SPSS for Windows 7.0. *p* < 0.05 was considered statistically significant.

## 3. Results

### 3.1. Protective Effects of DH Injection on Blood Flow Recovery, Collateral Vessel Formation, and Limb Function in Hind Limb Ischemia Diabetic Mouse Model

**Figure 3:**
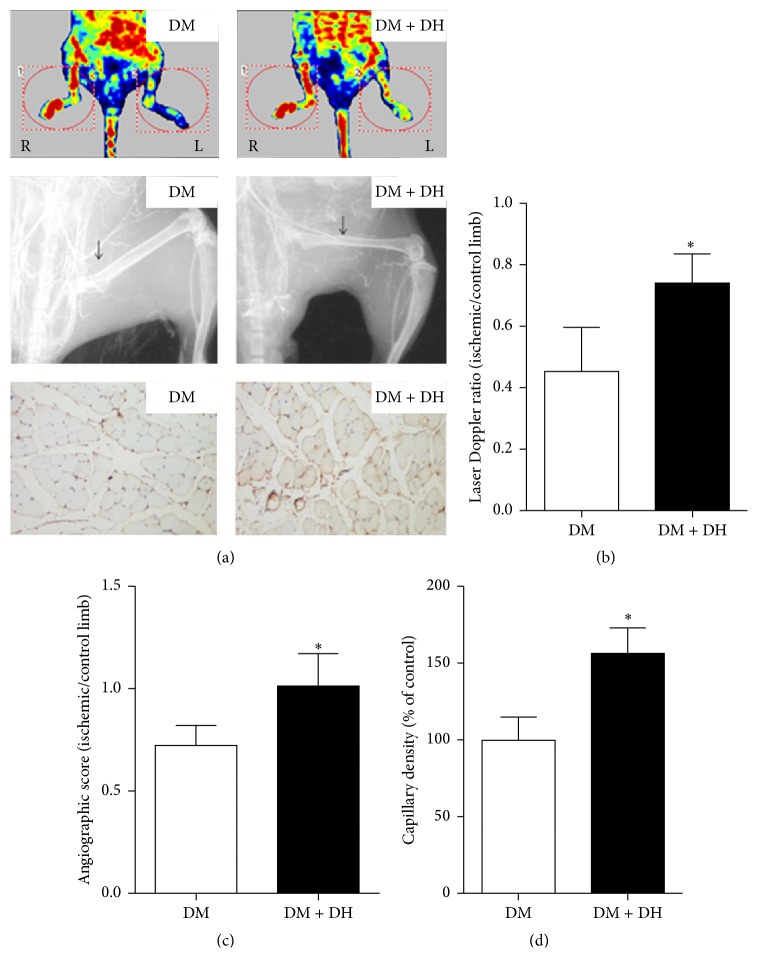
Effect of DH on recovery of blood flow monitored by LDPI, X-ray, and CD31 staining in diabetic mice. Representative evaluation of ischemic (right) and control (left) hind limbs on week 4 after operation. Blood flow perfusion was measured by LDPI, collateral circulation was assessed by angiography in X-ray, and CD31 staining was used to observe microvessel producing. DH significantly increased angiogenesis in diabetic hind limb ischemia versus DM model group (equal dosage PBS treatment). Data are expressed as mean ± SD. *∗* indicates *p* < 0.05 versus DM model group.

Perfusion recovery was significantly improved in mice receiving intraperitoneal DH administration at 2 mL/kg weight for 7 days as compared to control receiving equal amount of phosphate buffered saline as scheduled (Figures 3(a) and 3(b)).

Formation of collateral vessels below ligation site was examined using X-ray to ascertain whether improvement in tissue perfusion originated from increased blood flow or collateral vessel formation. At 2 weeks after ischemia induction, significantly more bridging collaterals with some degree of distal filling and originating from the internal iliac artery were visible in the thighs of mice treated with DH, in contrast to no apparent collateral vessel in the same area of controls (Figures 3(a) and 3(c)). In assessment of ischemic hind limbs using anti-mouse CD31 immunohistochemical staining, capillary density was significantly higher in DH i.p. than control group (Figures 3(a) and 3(d)).

**Figure 4:**
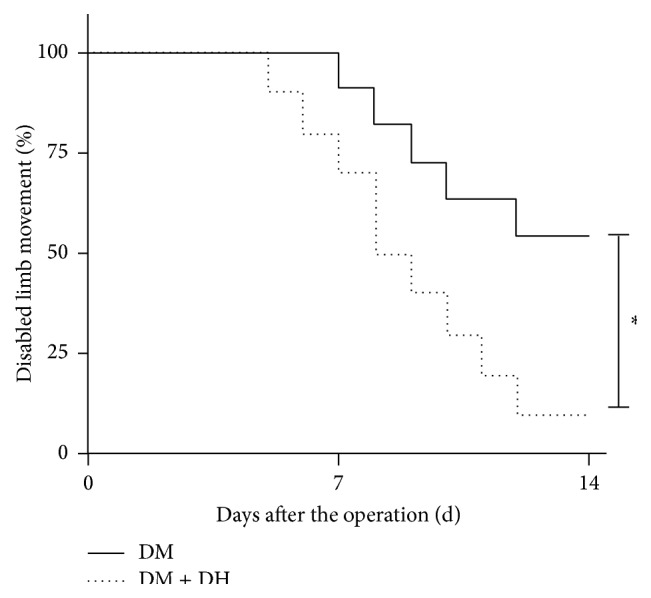
Effect of DH on ischemic limb function recovery. Representative serial evaluation of limb function in control DM model (full line) and DM + DH (dotted line) mice. Function was scored from 0 to 6, that is, from no movement to full and fast walking, and expressed as percentage of disabled limb movement (scores ≤ 3, supports weight, and may take 1 or 2 steps) for each day of 2-week experimental period. *∗* in vertical bar indicates *p* < 0.05 versus DM model group.

Survival analysis showed that after femoral artery ligation DH group mice recovered not only better but also faster than controls (Figure 4).

### 3.2. Activation of CSE-H_2_S-VEGF Axis in Association with DH Protective Effects

**Figure 5:**
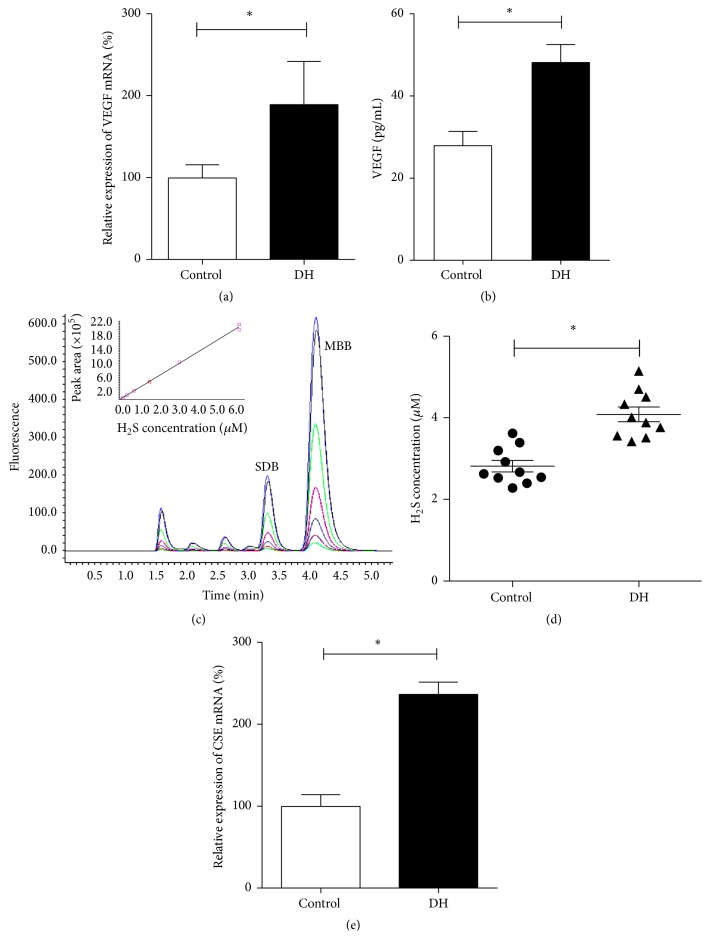
Activation by DH of CSE-hydrogen sulfide- (H_2_S-) VEGF axis in local tissue of diabetic mice. VEGF mRNA (a) and protein expression (b) in control and DH injected mice. H_2_S was analyzed by HPLC-FLD, and standard dilutions were used to plot the standard curve (*Y* (AUC) = 354000*X* (H_2_S, *μ*mol/L) + 7640; *R* ^2^ = 0.9996) (c). DH (triangles) versus control (circles) significantly increased H_2_S (d) and CSE (e) levels in femoral vein blood. Data are expressed as mean ± SD. *∗* indicates *p* < 0.05 versus control.

An antihyperglycemic effect of DH was excluded by lack of significant differences in weight (time 0: 30.61 ± 1.64 versus 30.39 ± 1.83 g, week 1: 31.23 ± 2.39 versus 31.02 ± 2.24 g, and week 2: 30.69 ± 2.43 versus 32.33 ± 3.18 g) and blood glucose levels (time 0: 6.66 ± 0.68 versus 6.26 ± 0.50 mmol/L, week 1: 11.32 ± 1.78 versus 10.05 ± 2.07 mmol/L, and week 2: 21.05 ± 1.64 versus 19.19 ± 1.67 mmol/L) between saline control and DH groups, respectively, during experimental period. CD31 levels in local muscular tissue increased after DH treatment, as did VEGF mRNA and protein levels compared with controls (Figures 5(a) and 5(b)). Several studies [18, 19] suggested a key role for muscular tissue H_2_S system in modulating VEGF expression after different stimuli, and DH was associated with increased venous H_2_S levels and CSE mRNA expression (Figures 5(c)–5(e)).

## 4. Discussion

This study showed that the traditional Chinese medicine herbal DH preparation improves blood flow in association with collateral vessel formation and activation of the cystathionine *γ*-lyase- (CSE-) hydrogen sulfide- (H_2_S-) vascular endothelial growth factor (VEGF) axis in a diabetic hind limb ischemia mouse model.

Ischemic vascular diseases remain a leading cause of mortality and morbidity worldwide [19] despite significant advances in medical and surgical intervention. Restoration of blood flow to ischemic organs is vital to prevent tissue death after arterial occlusion, and current treatment modalities are only partially efficacious. Discovery of angiogenic growth factors opened up the possibility of therapeutic angiogenesis for acute and chronic ischemia. In preclinical studies, three main approaches have been tested to deliver angiogenic agents: protein, gene, and cell therapy. In protein therapy, recombinant proteins are used directly to induce therapeutic effects [20], which are only transient because of the very short half-life of exogenous proteins in target tissues [21]. In contrast, gene therapy uses nonviral or viral vectors to carry a gene construct encoding a therapeutic protein into target tissues, where it is abundantly expressed [22]; however, this approach is hurdled by an inability to deliver genes efficiently and to obtain sustained expression. The premise of cell therapy in its present form is that transplanted cells by functioning as factories of multiple endogenous growth factors will induce vascular growth mainly in a paracrine manner rather than directly replacing damaged cells [23]. However, at least at the clinical level, efficacy of cell therapies has not been very satisfactory owing to poor in vivo viability of transfused cells, possibly reflecting abnormal cell microenvironment in pathological conditions.

A large body of evidence has shown the above limitations to current and developing therapeutic strategies apply to ischemic diseases associated with diabetes. Traditional Chinese medicine (TCM), widely used for centuries for ischemic diseases, has been the subject of numerous research reports elucidating effective components and their underlying mechanisms [24–28]. Exploring the effects of TCM on improvement of vascular microenvironment and functional recovery has significant clinical implications. Based on results from the present study, DH not only improved angiogenesis but also promoted ischemic organs' function recovery [29, 30], while our data are consistent with clinical experience and encourage clinical studies in the vast spectrum of diabetic patients. Association between local CSE-H_2_S-VEGF system and DH protective effects opens up the possibility that DH might act similar to H_2_S-donor drugs, which warrants further research.

DH treatment might have potential for use in other fields of medicine. However, proper assessment of therapeutic potential warrants further studies on mechanisms underlying DH protective effects such as the role of local tissue or bone marrow stem cells and in particular intracellular signaling pathways among others.
